# Giant Slip Induced Anomalous Dewetting of an Ultrathin Film on a Viscous Sublayer

**DOI:** 10.1038/s41598-017-14861-4

**Published:** 2017-11-07

**Authors:** Lin Xu, Dipankar Bandyopadhyay, Puchalapalli Dinesh Sankar Reddy, Ashutosh Sharma, Sang Woo Joo

**Affiliations:** 1 Laboratory of Surface Physics and Chemistry, Guizhou Education University, Guiyang, 550018 P. R. China; 2 Department of Chemical Engineering, Indian Institute of Technology Guwahati, Guwahati, 781039 India; 3 Department of Chemical Engineering, Indian Institute of Technology Kanpur, Kanpur, 208016 India; 4 School of Mechanical Engineering, Yeungnam University, Gyongsan, 712-749 South Korea

## Abstract

A ‘giant’ slip dynamics was engineered to a highly confined interface of a dewetting polymethylmethacrylate (PMMA) ultrathin film by introducing a lubricating viscous polystyrene (PS) sublayer. The crossover of regimes from no-slip to giant-slip was engendered by tuning the viscosity and thickness of the sublayer. A long-range hole-rim interaction with increase in slippage on the PMMA-PS interface transformed the circular holes on the PMMA surface into the noncircular faceted ones. The extent of the slippage and the transition of the length scales from slip-dominated to no-slip regime were evaluated using a general linear stability analysis. The proposed formulation provided an analytical tool to evaluate the slippage effective at the soft and deformable liquid-liquid interfaces.

## Introduction

Fluid dynamics in the mesoscales is often profoundly dissimilar from macroscopic ones due to the appearance of distinctive physics stemming from the highly confined boundaries^1,2^. For example, while the non-slipping flows manifesting from the viscous dissipations at the microscale irregularities near boundaries are rather common in the macroscopic domain^3–5^, slipping-boundaries are frequently encountered in micro or nanofluidic flows^6–8^. Furthermore, surfaces with marginal porosity, permeability, or roughness engender interesting boundary-driven flow properties to the super-fluids, ultrathin films (<100 nm), and flows on solvophobic surfaces^9–12^. Over the years, exploring the origins and the consequences of the slipping to non-slipping flows in various mesoscale systems have been an exciting subject of fundamental research. Herein, we exploit an anomalous dewetting behavior of an ultrathin polymer film stimulated by a highly confined viscous sublayer to correlate the experimental outcomes of the slip-induced dynamics with the theoretical ones. The experiments are designed to engineer tunable slippage at the confined boundary of a polymeric film, which leads to a morphological transition from circular to faceted non-circular holes on the film surface with increase in the slippage at the confined interface. A comprehensive theoretical framework has been developed to evaluate the crossover of regimes from no-slip to giant-slip domain^13,14^.

The dewetting of ultrathin polymer films could be significantly different in the no-slip^15–26^ and slip-dominated^27–38^ regimes. Previous studies showed that, on a non-slipping surface, the films dewetted to form circular holes with an average inter-hole spacing decided by the balance of stabilizing capillary and destabilizing van der Waals forces^15–26^. With time, these holes grew in size, and coalesced to form a ‘Voronoi tiling’ of polymer ribbons, which further underwent Plateau-Rayleigh instability to form a collection of randomly placed droplets. In comparison, introduction of slippage at the highly confined polymer-substrate rigid boundary was found to substantially change the dewetting dynamics. For example, the time for film-rupture was found to be much smaller in the slip regime as compared to the no-slip counterpart^30–39^. Furthermore, the holes grew faster (hole-radius, *R*: *t* ^2/3^) on a slipping surface as compared to the non-slipping one (*R*: *t*)^30–39^. Theoretically, Navier was the first to postulate the hydrodynamic slip boundary condition^40^ by correlating the tangential velocity (*u*) at the boundary to the shear rate as, ∂*u*/∂*z* = *u*/*b*, where *z* is the normal coordinate and the slip length, *b* = *μ*/*μ*_*s*_, is the ratio of the fluid viscosity (*μ*) to the surface friction coefficient (*μ*_*s*_). Later, the slip-induced flows were classified into four types based on this slip length, namely, non-slipping (*b* ~ 0), weakly slipping (*b ~* *h*), moderately slipping (*b* > *h*), and strongly slipping (*b ≫* *h*) regimes^30–39^. Apart from the formation of holes and kinetics of their growth, the dewetted morphologies were found to be significantly different in the various slip regimes. For example, high-molecular-weight polymer films showed a substantially large hole-spacing (*L*) with increase the slippage at the boundary when the film thickness was kept constant. In comparison, when *L* was plotted against the change in the thickness (*L*∞*h* ^*n*^) in a log-log scale the change in the slope was found to be non-monotonic in the strongly (*n* = 1.75), weakly (*n* = 1.25), and no slip regimes (*n* = ~2)^39^. Importantly, previous works indicate that, although the slip boundaries have significant influence on various scientific and technologically important prototypes, there are very few experimental or theoretical framework available for the analysis of the slippage in the micro or nanoscale flows. In particular, quantitative measurement of slippage at the deformable liquid-liquid interface^13,14^ is perhaps among one of the most neglected areas of research.

**Figure 1:**
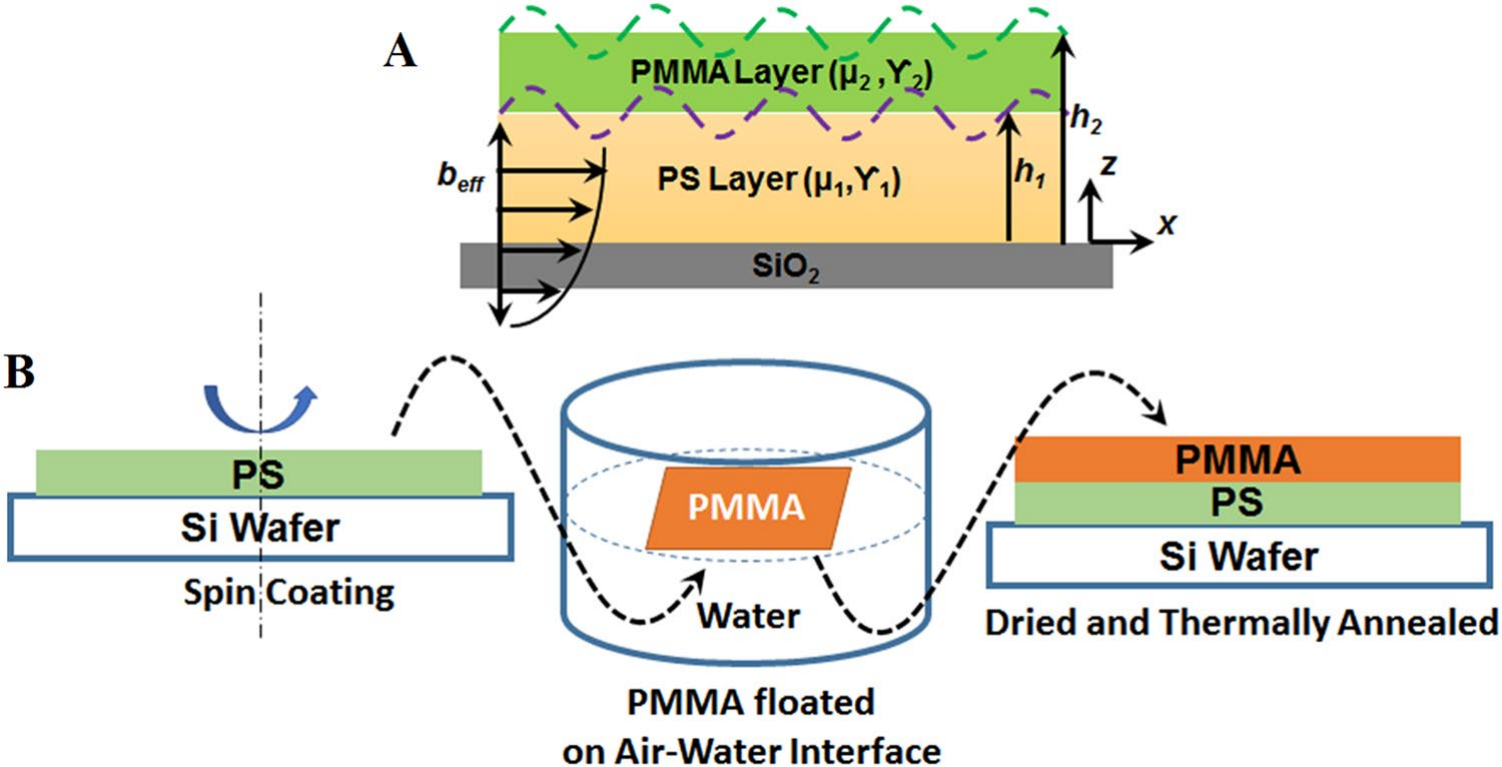
(**A**) Schematic representation of a PMMA/PS/SiO_2_ bilayer. The PMMA film and the combined PS/PMMA film thicknesses are denoted by *h*_1_ and *h*_2_. The symbols *μ*_*i*_ and *γ*_*i*_ denote the viscosity and surface tension of the respective layers. The notation *b*_*eff*_ shows the effective slip-length at the PS-PMMA interface. (**B**) Schematically shows the steps for the bilayer preparation for the dewetting experiments.

In this context, the present study unveils an experimental cum theoretical approach to analyze the dynamics of an ultrathin film undergoing slippage. The proposed framework is multipurpose in the sense that the dynamics of slipping to non-slipping ultrathin films can be analyzed on the rigid boundaries as well as on the deformable interfaces. Figure 1A schematically shows the prototype where an ultrathin poly-methylmethacrylate (PMMA) layer dewetted on a polystyrene (PS) sublayer which in turn was coated over a silica (SiO_2_) substrate. A highly viscous (higher molecular weight - Mw) and thin PS sublayer ensured that a highly viscous and thin PMMA layer dewetted on a nearly rigid non-slipping surface whereas a thick and a low viscosity (low Mw) PS sublayer facilitated a ‘giant-slip’ induced dynamics to the same PMMA layer through the deformable PS-PMMA interface. Figure 1B schematically shows the steps for the bilayer preparations for the dewetting experiments. A morphological transition from circular to faceted non-circular holes was observed, while the flow dynamics of the polymer film was changed from no-slip (*b*_*eff*_ ~ 0) to the giant-slip (*b*_*eff*_ ~ mm) regime.

Apart from the shape distortion, the average hole-spacing at the PMMA layer was also found to increase with the enhancement of slippage at the PS-PMMA boundary. The change of slippage was theoretically estimated thorough a modified Navier’s slip boundary condition, which was formulated from a general linear stability analysis (GLSA) of the governing equations and boundary conditions. Previous studies indicated that such bilayer configurations could be unstable when the destabilizing van der Waals force was strong enough to deform the soft polymer-polymer and/or polymer-air interface(s)^41–50^. In particular, the hole formed on the upper layer was found to grow explosively (hole-radius, *R ~* *t* ^*0.71*^) with the reduction in the viscosity or Mw of the polymer sublayer and subsequent increase in the slippage at the interface^49,50^. However, the slippage at the confined PS-PMMA interface of such configurations could not be analyzed solely from the simple Navier’s condition because it required the continuity of velocities alongside the balances of the normal and the tangential stresses to be satisfied. In the proposed methodology, we evaluated the effective slip length (Fig. 1, *b*_*eff*_) at the PS-PMMA interface as the ratio of the tangential velocity to the total shear rate, which was a measure of the energy loss through viscous dissipation at the PS-PMMA interface.

## Results and Discussion

**Figure 2:**
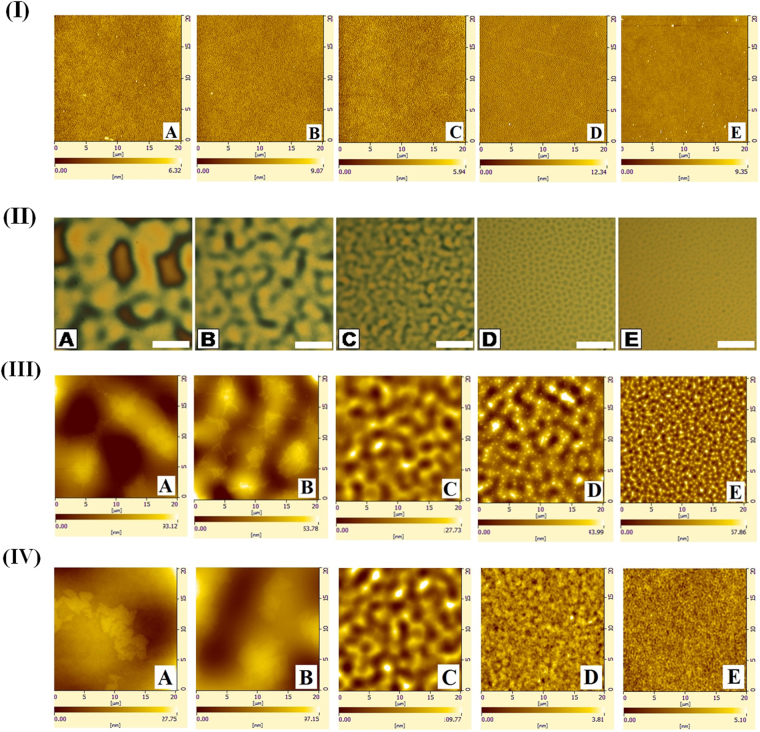
A series of AFM images [row (I)] show the morphologies of the PMMA-air interface with varying Mw of the PS film before annealing. A series of optical micrographs [row (II)] and AFM images [row (III)] illustrating the morphologies of the PMMA-air interface with varying Mw of the PS film when heated to 160 °C. Images (**A**–**E**) in both the rows correspond to PS Mw = 13 kg/mol (annealing time: 1.5 min), 31 kg/mol (annealing time: 5 min), 97 kg/mol (annealing time: 20 min), 390 kg/mol (annealing time: 180 min) and 2000 kg/mol (annealing time: 240 min), respectively, when PMMA Mw = 50 kg/mol (1.75 × 10^7^ Pa s)^51–53^, *h*_*PMMA*_ = 18 ± 2 nm and *h*_*PS*_ = 174 ± 5 nm. A series of AFM images [row (IV)] show the morphologies of the PMMA-air interface with varying Mw of the PS film when heated to 160 °C for the same systems in row (II and III) with an annealing time of 20 min. The size of the bar on the optical micrographs is 20 µm.

A series of AFM images in the row (I) of the Fig. 2 show the morphologies of the PMMA-air interface with varying Mw of the PS film before annealing. The image confirmed that the films were homogeneous and flat surface after bilayer preparation and dewetted only after annealing. The optical micrographs [row (II)] and the AFM images [row (III)] in the Fig. 2 show the morphologies of the PMMA-air interface at the initial stage of dewetting as the Mw of the PS sublayer was varied to modulate the friction at the PS-PMMA interface. In the experiments the PMMA and PS layer thicknesses were kept constant at 18 ± 2 nm and 174 ± 5 nm, respectively.

A high Mw PMMA layer ensured that the viscosity of the upper film remains very high^51–53^. While the optical micrographs shown in this figure depict the hole formation stage, the AFM images show the early stage of surface undulations of the PS film confirming the onset of *spinodal* dewetting. Image (IA) shows that a thin and highly viscous PMMA layer dewetted with very large hole-spacing on a thick and less viscous low Mw PS sublayer. Interestingly, in such a configuration, the circular holes at the initial stages of dewetting were found to quickly deform into the faceted non-circular ones, as shown in the image (IA). This is in stark contrast to the dewetting of a non-slipping PMMA film on a rigid PS surface where the holes preserved the circular shapes until the hole-rims started coalescing with each other. Images (B) – (E) in the row I show the change in the hole-morphologies on the highly viscous PMMA surface with increase in the viscosity at the PS sublayer. These images suggest that with the increase in the viscosity or Mw of the PS sublayer, the PS-PMMA interface behaved more like a solid surface, which led to the formation of the circular holes with a much smaller lateral spacing. A series of AFM images in the row (IV) show the morphologies of the PMMA-air interface with varying Mw of the PS film when heated to 160 °C for the same systems as shown in the rows (II) and (III), however, after a fixed annealing time of 20 min. Again, these images also suggest that there was an increase in the spinodal length scale as the PS layer became more viscous at lower Mw.

The experiments also revealed that the hole-rims of the slipping PMMA film were under a long-range interaction even at the hole-formation stage due to a faster kinetics of hole-growth (*R* ∞ *t* ^0.71^)^49,50^ as compared to the non-slipping scenario (*R* ∞ *t*). The long-range interactions between the hole-rims were strong enough to influence the growth of the sparsely populated holes (image IA, Fig. 2) on the slipping PMMA layer as compared to the more frequently spaced (image IE, Fig. 2) holes in the non-slipping regime. A faster long-range interaction of the hole-rims of the slipping PMMA layer grew faster (slower) in the directions where the interactions between the different hole-rims were less or absent (present). This anisotropic hole-growth due to the long-range hole-rim interaction between the randomly placed holes led to the formation of the faceted holes with non-circular shape. Remarkably enough, although the thermodynamic parameters such as surface tension and van der Waals forces could partially influence the hole-formation stage, the anisotropic hole-growth was dictated by the kinetics due to the slippage at the PS-PMMA interface.

**Figure 3:**
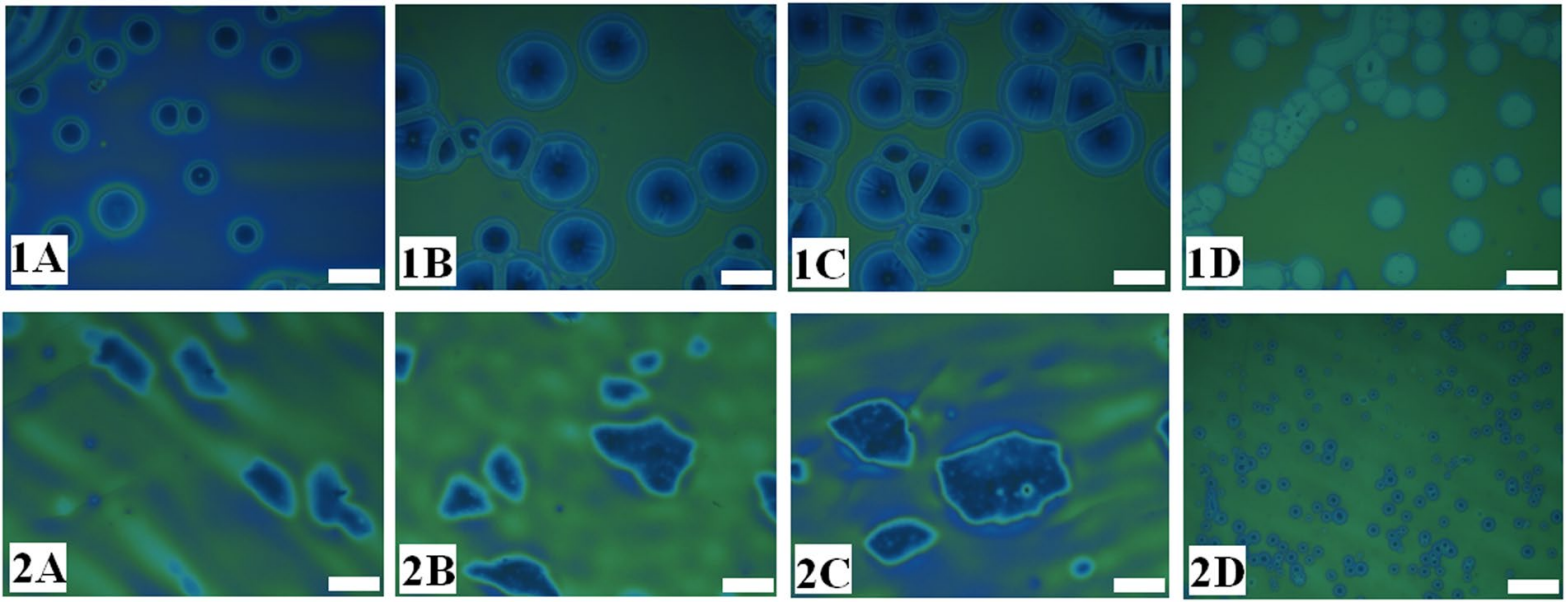
Optical micrographs illustrating the morphology of the PMMA-air interface when the annealing temperature was 180 °C. In image sets 1 and 2, PMMA Mw = 10 kg/mol (3.1 × 10^3^ Pa s) and 350 kg/mol (5.51 × 10^8^ Pa s)^51–53^, respectively, when *h*_*PMMA*_ **= **35 ± 2 nm and *h*_*PS*_ = 174 ± 5 nm. Images (**A**–**D**) in both the sets correspond to PS Mw = 4 kg/mol, 13 kg/mol, 31 kg/mol, and 2000 kg/mol, respectively. The size of the bar is 40 µm.

Importantly, the experiments also showed that when the viscosity contrast across the PS-PMMA interface was reduced, the transition from non-circular to circular morphologies could again be observed. In Fig. 3 the image sets 1 and 2 represent PMMA film having two different viscosities (Mw). The images (A–D) in each set show the short-time morphologies for the bilayer having PS sublayer of different viscosities (Mw). Comparing the morphologies in the set 1 and 2, we could conclude that when the PMMA film was less viscous and the viscosity-contrast across the interface was less, the dewetting progressed following the non-slipping way. In such a scenario, the holes grew while preserving the circular shape as shown by images 1A–1D. In comparison, when a highly viscous PMMA layer dewetted on a less viscous and more lubricating PS sublayer, the faceted non-circular morphologies emerged during the hole-growth stage, as shown by the images 2A–2C in the Fig. 3. In contrast, image 2D in the Fig. 3 showed that when the viscosity at PS sublayer was substantially high, again the hole-rims showed frequently spaced circular holes, a non-slipping behavior.

**Figure 4:**
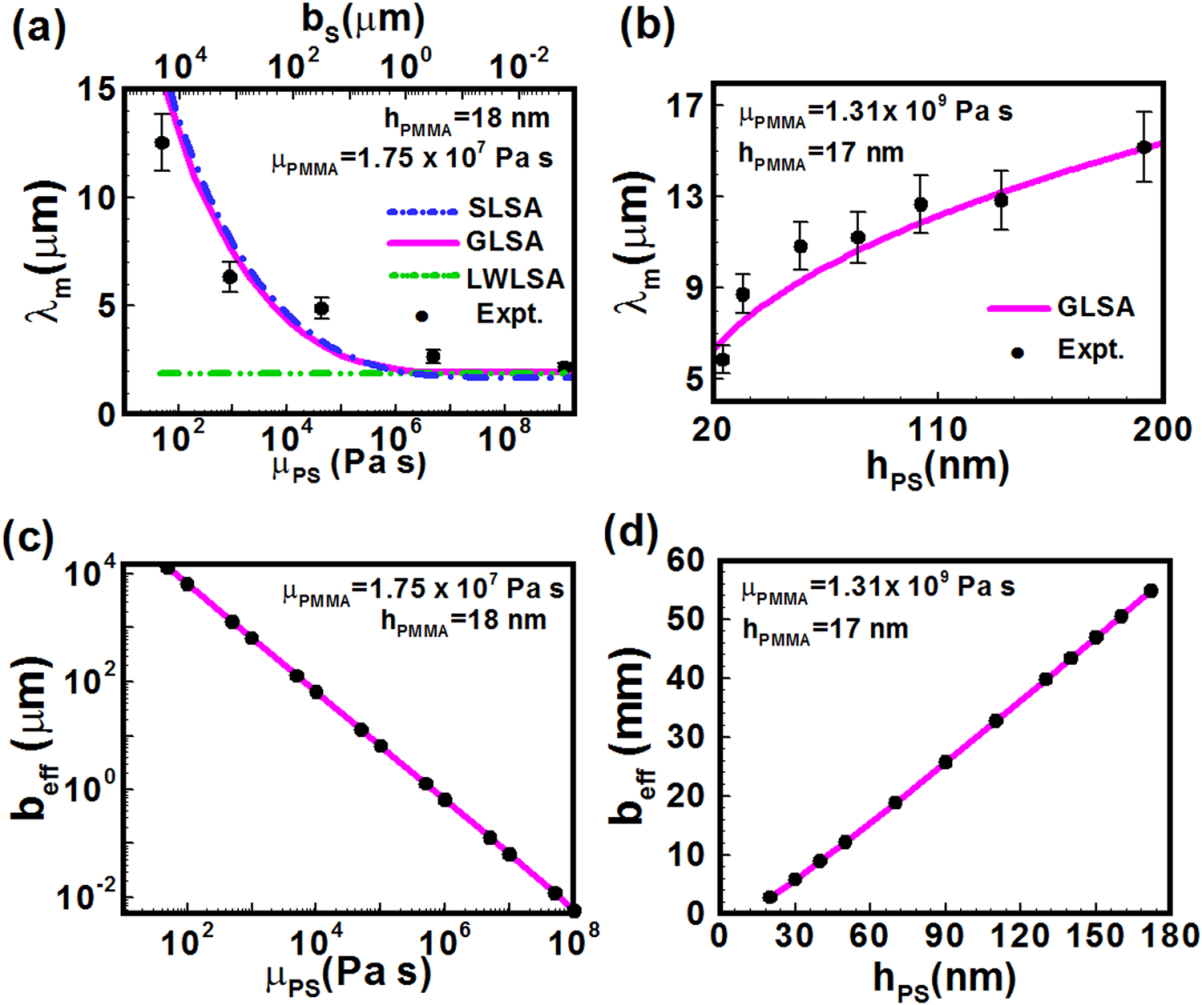
Plots (**a**) and (**b**) show a comparison between the experiments (filled circles) and *λ*_*m*_ from the LSA (dash-dot-dot line – LWLSA, and solid line – GLSA) with the variations in the viscosity $({\mu }_{PS}\equiv {\mu }_{1})$ and thickness $({h}_{PS}\equiv {h}_{1})$ of the PS layer. The dash-dot line in plot (**a**) also shows the variation in *λ*_*m*_ with slip-length $({b}_{s})$ from SLSA when the PMMA layer was strongly slipping layer on a rigid PS surface. In the plots (**c**) and (**d**) the variation in the effective slippage $({b}_{eff})$ at the PS-PMMA interface evaluated from the GLSA is shown with the variations in ${\mu }_{PS}$ and ${h}_{PS}$. The other parameters were, ${\gamma }_{PS}$ = 0.03 N/m, ${\gamma }_{PMMA}$ = 0.04 N/m, and ${\gamma }_{Si{O}_{2}}$ = 0.062 N/m; in the plots (**a**) and (**c**) *h*_*PS*_ = *h*_10_ = 172 nm, *h*_*PMMA*_ = *h*_20_ - *h*_10_ = 18 nm, ${\mu }_{PMMA}\equiv {\mu }_{2}$ = 1.75 × 10^7^ Pa s; in the plots (**b**) and (**d**) *h*_*PMMA*_ = 17 nm, ${\mu }_{PMMA}\equiv {\mu }_{2}$ = 1.75 × 10^7^ Pa s, and ${\mu }_{PS}\equiv {\mu }_{1}$ = 890 Pa s^51–53^. For the SLSA curve in plot (**a**) ${\gamma }_{eff}$ was set to 0.000717 N/m. For the experimental results the annealing temperature was 160 °C. In plot (**b**) the viscosities of the PMMA (Mw = 350 kg/mol) and PS (Mw = 31 kg/mol) layers were kept constant by fixing Mw^51–53^.

The slip-induced instabilities in the PS-PMMA bilayers discussed above also led to the change in length scales. The discrete points in Fig. 4a shows that the average spacing between the holes grew as the viscosity (Mw) of the PS sublayer was progressively reduced to increase the slippage at the PS-PMMA interface. A similar trend was also observed in Fig. 4b when the PS film thickness was changed from thin to thick and the amount of slippage at the PS-PMMA interface was increased by providing more lubrication through the PS sublayer. Figure 4b shows that for a configuration with high viscosity PMMA layer as the thickness of the PS sublayer was progressively increased, the lubrication under the PMMA layer was also enhanced which in turn increased the hole-spacing. However, the trends in the Fig. 4a and b were not found to be similar because in the latter case the change in thickness also altered the strength of the van der Waals forces, which contributed to the change in the length scale alongside the slippage.

The experimental results summarized in the Fig. 4 were fitted against the theoretical model developed for the prediction of slippage, which for the sake of brevity is provided later in the methods section. In the theoretical model, both the films were assumed to be Newtonian and incompressible fluids and were unstable by the van der Waals forces while the surface and the interfacial tensions acted as the stabilizing influences. The correlation between the Mw and film viscosities was obtained from the previous works^51–53^. Further, since in most of the experiments the PS layer was kept ~20 nm, we assumed that the dewetting followed solely the *spinodal* pathway in which the surface initial surface undulations grew to form the holes. Thus, the length scale obtained from the GLSA was correlated with the hole spacing of the initial stages *spinodal* dewetting. In Fig. 4a and b, the solid lines show the transition from smaller to larger wavelength with reduction in the viscosity or increase in thickness of the PS sublayer could be quantitatively predicted by the GLSA from the dispersion relation Eq. 1. The dash-dot-dot line in figure shows that the predictions from the long-wave dispersion relation (LWLSA), which was unable to capture the slip dominated transition from smaller to longer wavelength regime. The point of deviation of the LWLSA from the GLSA was found to be the zone where the transition from non-slipping to slip-induced regime took place, which was also marked by the appearance of non-circular faceted holes in the experiments.

We evaluated the effective slippage (*b*_*eff*_) at the PS-PMMA interface from the GLSA as the ratio of the tangential velocity to the total shear rate at the PS-PMMA interface, as later described in the problem formulation section. For this purpose, initially, the tangential component of the hydrodynamic stress at the PS-PMMA interface, $({u}_{2z}+{w}_{2x})(1-{h}_{1x}^{2})+2{h}_{1x}({w}_{2z}-{u}_{2x})$, was linearized to the expression, $d{\tilde{u}}_{2}={(-ik)}^{-1}{\tilde{w}}_{2zz}+ik{\tilde{w}}_{2}$ and then the effective slippage at the PS-PMMA interface (*h*_1_) was evaluated as, ${b}_{eff}={\tilde{u}}_{2}/d{\tilde{u}}_{2}$, employing the physical properties, dominant growth coefficient (*ω*_*m*_) and the corresponding wavelength (*λ*_*m*_) for a given PS-PMMA configuration. Here *h*_1_ and *h*_1_ are the thicknesses of the films as depicted in the Fig. 1, *u*_*i*_ and *w*_*i*_ are the *x*- and *y*-directional velocities and the variables with over-tilde symbols represent the perturbed variables. The subscripts ‘*x*’ and ‘*z*’ in the expressions represent the derivatives. The aforementioned method was found to be more comprehensive in predicting slippage at the deformable PS-PMMA interfaces than the commonly employed Navier’s method^40^. Figures 4c and d show that variation in *b*_*eff*_ with the viscosity and the thickness of the PS sublayer, respectively. The plots suggest a transition from non-slipping to ‘giant’ slipping (*b*_*eff*_ ~mm) regimes took place when a highly viscous ultrathin PMMA film dewetted on a low to high viscosity PS sublayer. When the PS sublayer was thin and highly viscous, it behaved more like a rigid and non-deformable surface where PMMA film could show a non-slipping dewetting behavior. In contrast, a thick and low viscosity PS sublayer could stimulate a ‘giant’ slippage at the PS-PMMA interface for a highly viscous PMMA layer. Importantly, the dispersion relation for a strongly slipping single layer (SLSA – Eq. (2) or Eq. (8) of ref.^9^) derived using Navier’s condition could also heuristically predict the GLSA or experimental values when interfacial tension of the PS-PMMA interface was employed in place of surface tension and the slip length was varied from 10^−2^ μm–10^4^ μm, as shown in the Fig. 4a.

## Conclusions

In summary, we demonstrated that highly viscous PMMA layers showing anomalous non-circular morphologies after dewetting, while they experienced ‘giant’ slippage on the less viscous and thick PS sublayers. The transition from circular to non-circular morphology was observed due to a long-range interaction between the fast-moving hole-rims under the influence of slippage at the PMMA-PS interface. The interaction ensured an anisotropic hole-growth in the directions where they could sense the absence of the other hole-rims. The slip-dominated faceted non-circular holes had a larger average inter-hole distance, which was predicted accurately by a general linear stability analysis. The theoretical formulation provided an analytical way to evaluate the effective slippage at the PMMA-PS interface of such a complex configuration, proving prediction of the regime crossover from no-slip to giant slip.

## Methods

### Experimental Section

For the experiments the PMMA-PS-SiO_2_ bilayers were prepared in the following manner. PS films (Mw = 4 kg/mol, 13 kg/mol, 31 kg/mol, 97 kg/mol, 390 kg/mol and 2 000 kg/mol; Mw/Mn < 1.1) were spin-coated from a toluene solution on the Si wafer with a thick oxide layer. PMMA films (Mw = 10 kg/mol, 50 kg/mol, 350 kg/mol; Mw/Mn < 2) were spin-coated from a chloroform solution onto mica, and subsequently floated onto the surface of de-ionized water and deposited on the PS layer to form a bilayer. Figure 1B schematically shows the steps for the bilayer preparations for the dewetting experiments. The thicknesses of the films were measured employing ellipsometry (MM-16, HORIBA Jobin Yvon, France). The residual solvent was removed by putting the films in a vacuum oven for 24 h at room temperature. After drying, the films were annealed at a temperature well above the glass transition of both the polymers to engender dewetting^54^. The surface morphology was observed by optical microscopy (OM, ZEISS Microsystems, Germany) in reflection mode with an attached CCD camera and atomic force microscopy (AFM, Seiko Instruments Inc., Japan) operating in the tapping mode. The spring constant of the cantilever is 2 N/m. The cantilever oscillated close to its resonance frequency between 65 and 75 kHz.

### Problem Formulation

The change in the spacing between the holes with the change in the Mw and thickness of the lower layer was explained employing a general linear stability analysis applicable for all wavenumbers. We assumed that the PS and PMMA films were Newtonian liquids,${{\bf{T}}}_{i}={\mu }_{i}(\nabla {{\bf{u}}}_{i}+\nabla {{\bf{u}}}_{i}^{T})$, above their glass transition temperature and the variation in the Mw changed the viscosity of the PS film^51–53^. Further, the inertial terms were neglected from the equations of motion because of the thinness and high viscosity of the films. Thus, the equations of motion in the Stokes flow regime, $-\nabla {P}_{i}+\nabla \cdot {{\bf{T}}}_{i}=0$, together with the continuity equation for the incompressible films, $\nabla \cdot {{\bf{u}}}_{i}=0$, described the dynamics of the viscous bilayer, as shown in Fig. 1. The subscript *i* denoted the lower PS layer (*i* = 1) and the upper PMMA layer (*i* = 2). For the *i* ^th^ layer: ${{\bf{T}}}_{i}$, ${{\bf{u}}}_{i}\{{u}_{i},{w}_{i}\}$, ${\mu }_{i}$, ${\gamma }_{i}$, ${\pi }_{i}$, ${p}_{i}$, and ${P}_{i}$ ($={p}_{i}-{\pi }_{i}$) denoted the stress tensor, velocity vector, viscosity, excess pressure because of intermolecular forces, static pressure in the liquid and the effective non-body force pressure inside the films, respectively.

The lower PS sublayer was assumed to be non-slipping and non-permeating, (**u**_1_ = 0), through the SiO_2_ surface, at *z* = 0. At the confined PS-PMMA interface (*z* = *h*_1_), continuity of *x*- and *z*-components of velocities, (**u**_1_ = **u**_2_), normal, ($-{p}_{2}+{{\bf{n}}}_{1}\cdot {{\bf{T}}}_{2}\cdot {{\bf{n}}}_{1}+{p}_{1}-{{\bf{n}}}_{1}\cdot {{\bf{T}}}_{1}\cdot {{\bf{n}}}_{1}={\gamma }_{21}{{\boldsymbol{\kappa }}}_{1}$), and tangential, (${{\bf{t}}}_{1}\cdot {{\bf{T}}}_{2}\cdot {{\bf{n}}}_{1}={{\bf{t}}}_{1}\cdot {{\bf{T}}}_{1}\cdot {{\bf{n}}}_{1}$), stress balance equations, and the kinematic condition, ${\dot{h}}_{1}+({u}_{1}\partial {h}_{1}/\partial x)={w}_{1}$, were enforced as boundary conditions. In addition, the normal, ($-{p}_{2}+{{\bf{n}}}_{2}\cdot {{\bf{T}}}_{2}\cdot {{\bf{n}}}_{21}=-{\gamma }_{2}{{\boldsymbol{\kappa }}}_{2}$) and tangential stress balances **t**_2_ · **T**_2_ · **n**_2_, and the kinematic condition, ${\dot{h}}_{2}+({u}_{2}\partial {h}_{2}/\partial x)={w}_{2}$, were enforced as boundary conditions at the PMMA-air free-surface (*z* = *h*_2_). The symbols **n**_**i**_ and **t**_**i**_ in the boundary conditions represented unit normal, $(-{h}_{ix}/\sqrt{(1+{h}_{ix}^{2})},1/\sqrt{(1+{h}_{ix}^{2})})$, and tangent, $(1/\sqrt{(1+{h}_{ix}^{2})},{h}_{ix}/\sqrt{(1+{h}_{ix}^{2})})$ vectors, respectively. The superscript dot and subscript *x* in the expressions represented the time and space derivatives of the variables. The variable thicknesses of the bilayer and of the lower layer were represented by *h*_1_ and *h*_2_, respectively. The symbols *h*_10_ and *h*_20_ represented the respective base state thicknesses. The excess pressures because of the intermolecular forces at the interfaces were, ${\pi }_{1}=-{A}_{1}/6\,\pi \,{h}_{1}^{3}-{A}_{2}/6\,\pi \,{h}_{2}^{3}$ and ${\pi }_{2}=-{A}_{3}/6\,\pi \,{({h}_{2}-h1)}^{3}-{A}_{2}/6\,\pi \,{h}_{2}^{3}$ ^45–47,55,56^. The disjoining pressures were written in terms of effective Hamaker constants, which were derived from the binary Hamaker constants, ${A}_{1}={A}_{11}+{A}_{S2}-{A}_{S1}-{A}_{12}$_,_ ${A}_{2}={A}_{12}-{A}_{s2}$ and ${A}_{3}={A}_{22}-{A}_{12}$. The *s*, 1, and 2 in the binary Hamaker constants represented the solid substrate, lower and upper layer, respectively.

### General Linear Stability Analysis

In order to perform general linear stability analysis (GLSA), we linearized the governing equations using the normal linear modes, ${{\bf{u}}}_{i}={\tilde{{\bf{u}}}}_{i}{e}^{\omega t+{\bf{i}}kx}$, ${P}_{i}={\tilde{P}}_{i}{e}^{\omega t+{\bf{i}}kx}$ and ${h}_{i}={h}_{i0}+{\tilde{\delta }}_{i}{e}^{\omega t+{\bf{i}}kx}$ where the symbols −*ω* and *κ* represented the linear growth coefficient and the wave number of disturbance, respectively. The perturbed variables were represented by the over-tilde symbols. Eliminating ${\tilde{P}}_{i}$ from the linearized governing equations led to, ${\tilde{w}}_{izzzz}-2{k}^{2}{\tilde{w}}_{izz}+{k}^{4}{\tilde{w}}_{i}=0$, a biharmonic equation for each layers having a general solution, $\tilde{w}=({B}_{1i}+{B}_{2i}z){e}^{kz}+({B}_{3i}+{B}_{4i}z){e}^{-kz}$. The boundary conditions were also linearized to the following forms, (i) at the PS-SiO_2_ interface (*z* = 0) no-slip and impermeability conditions (${\tilde{u}}_{1}$ = ${\tilde{w}}_{1}$ = 0) were enforced; (ii) at the PS-PMMA interface (*z* = *h*_10_) continuity of velocities, ${\tilde{u}}_{1}$ = ${\tilde{u}}_{2}$ and ${\tilde{w}}_{1}$ = ${\tilde{w}}_{2}$, tangential stress balance, ${\mu }_{1}({\tilde{u}}_{1z}+ik{\tilde{w}}_{1})={\mu }_{2}({\tilde{u}}_{2z}+ik{\tilde{w}}_{2})$, normal stress balance ${\tilde{P}}_{1}-{\tilde{P}}_{2}-2{\mu }_{1}{\tilde{w}}_{1z}+2{\mu }_{2}{\tilde{w}}_{2z}+{\omega }^{-1}[{|(-{k}^{2}{\gamma }_{21}+{\pi }_{1{h}_{1}}-{\pi }_{2{h}_{1}}){\tilde{w}}_{1}|}_{{h}_{10},{h}_{20}}+{|({\pi }_{1{h}_{2}}-{\pi }_{2{h}_{2}}){\tilde{w}}_{2}|}_{{h}_{10},{h}_{20}}]=0$, and kinematic condition, ${\tilde{\delta }}_{1}={|{\tilde{w}}_{1}|}_{{h}_{10}}/\omega$ were enforced; (iii) at the PMMA-air interface (*z* = *h*_20_), the tangential, ${\mu }_{2}({\tilde{u}}_{2z}^{x}+ik{\tilde{w}}_{2})=0$, and normal, ${\tilde{P}}_{2}-2{\mu }_{2}{\tilde{w}}_{2z}+{|(-{k}^{2}{\gamma }_{2}+{\pi }_{2{h}_{2}}){\tilde{w}}_{2}|}_{{h}_{10},{h}_{20}}+{|{\pi }_{2{h}_{1}}{\tilde{w}}_{1}|}_{{h}_{10},{h}_{20}}=0$, stress balances and the kinematic condition, ${\tilde{\delta }}_{2}={|{\tilde{w}}_{2}|}_{{h}_{20}}/\omega$ were enforced. Here ${\tilde{u}}_{i}$ and ${\tilde{w}}_{i}$ were the *x-* and *z*-components of perturbed velocities, ${h}_{10}$ and ${h}_{20}$ were base state PS film and combined PS/PMMA film thicknesses, ${a|}_{b}$ denoted that the variable *a* evaluated under the condition *b*, and subscripts *x*, *z*, ${h}_{1}$, and ${h}_{2}$ denoted ordinary differentiations.

1 $$|\begin{array}{cccccccc}1 & 1 & 0 & 0 & 0 & 0 & 0 & 0\\ 1 & -1 & \frac{1}{k} & \frac{1}{k} & 0 & 0 & 0 & 0\\ {J}_{1} & {J}_{2} & {h}_{1}\,{J}_{1} & {h}_{1}\,{J}_{2} & -\,{J}_{1} & -\,{J}_{2} & -\,{h}_{1}\,{J}_{1} & -\,{h}_{1}\,{J}_{2}\\ -\,{J}_{1} & {J}_{2} & -\frac{{J}_{1}{J}_{5}}{k} & \frac{{J}_{2}{J}_{6}}{k} & {J}_{1} & -{J}_{2} & \frac{{J}_{1}{J}_{5}}{k} & \frac{{J}_{2}{J}_{6}}{k}\\ 2k\,{\mu }_{1}{J}_{1} & 2k\,{\mu }_{1}{J}_{2} & 2\,{\mu }_{1}\,{J}_{1}{J}_{5} & 2\,{\mu }_{1}{J}_{2}{J}_{6} & -2k\,{\mu }_{2}{J}_{1} & -2k\,{\mu }_{2}{J}_{2} & -2{\mu }_{2}\,{J}_{1}{J}_{5} & -2{\mu }_{2}\,{J}_{2}{J}_{6}\\ 0 & 0 & 0 & 0 & 2k\,{\mu }_{2}{J}_{3} & 2k{\mu }_{2}{J}_{4} & 2{\mu }_{2}{J}_{7}{J}_{3} & 2{\mu }_{2}{J}_{8}{J}_{4}\\ {J}_{1}{J}_{9} & {J}_{2}{J}_{10} & {h}_{1}{J}_{1}{J}_{9} & {h}_{1}{J}_{2}{J}_{10} & {J}_{11} & {J}_{12} & {J}_{13} & {J}_{14}\\ {J}_{1}{\varphi }_{3}/\omega  & {J}_{2}{\varphi }_{3}/\omega  & {h}_{1}{J}_{1}{\varphi }_{3}/\omega  & {h}_{1}{J}_{2}{\varphi }_{3}/\omega  & {J}_{3}{J}_{15} & {J}_{4}{J}_{16} & {h}_{2}{J}_{3}{J}_{15} & {h}_{2}{J}_{4}{J}_{16}\end{array}|=0$$

where $${\varphi }_{1}=\partial {\pi }_{1}/\partial {h}_{1}-\partial {\pi }_{2}/\partial {h}_{1};\,{\varphi }_{2}=\partial {\pi }_{1}/\partial {h}_{2}-\partial {\pi }_{2}/\partial {h}_{2};\,{\varphi }_{3}=\partial {\pi }_{2}/\partial {h}_{1};\,{\varphi }_{4}=\partial {\pi }_{2}/\partial {h}_{2};\,{J}_{2}={e}^{-k{h}_{1}}$$ $${J}_{3}={e}^{k{h}_{2}};$$ $${J}_{4}={e}^{-k{h}_{2}};{J}_{5}=1+k{h}_{1};{J}_{6}=-1+k{h}_{1};{J}_{7}=1+k{h}_{2};{J}_{8}=-1+k{h}_{2};{J}_{9}=(-2k\omega {\mu }_{1}+{\varphi }_{1})/\omega ;$$ $${J}_{10}=(2k\omega {\mu }_{1}+$$ $${\varphi }_{1})/\omega ;{J}_{11}=2k{\mu }_{2}J{}_{1}+J{}_{3}\varphi _{2}/\omega ;{J}_{12}=-2k{\mu }_{2}J{}_{2}+J{}_{4}\varphi _{2}/\omega ;{J}_{13}=2kh{}_{1}\mu _{2}J{}_{1}+h{}_{2}J{}_{3}\varphi _{2}/\omega ;$$ $${J}_{14}=-2kh{}_{1}\mu _{2}J{}_{2}+h{}_{2}J{}_{4}\varphi _{2}/\omega ;$$ $${J}_{15}=(-2k\omega {\mu }_{2}+{\varphi }_{4})/\omega ;\,{J}_{16}=(2k\omega {\mu }_{2}+{\varphi }_{4})/\omega $$.

Replacing the expressions for the perturbed variables ${\tilde{u}}_{i}$, ${\tilde{w}}_{i}$ and ${\tilde{P}}_{i}$ in the linearized boundary conditions led to a set of eight homogeneous linear algebraic equations involving eight unknown constants, *B*_*ji*_ (*j* = 1 to 4 and *i* = 1, 2). The general dispersion relation Eq. (1) was obtained by equating the determinant of the coefficient matrix of the set of algebraic equation to zero. The solution of the Eq. (1) was an analytical expression involving the linear growth coefficient and the wave number [$\omega =f(k)$] when the physical properties and the thicknesses of the films were known. The dominant growth coefficient (${\omega }_{m}$) and the corresponding wavelength (${\lambda }_{m}$) were obtained by finding the global maxima of $\omega$ and the corresponding wavelength from the dispersion relations. The general dispersion relation could be reduced to the long-wave dispersion relation (LWLSA)^45–47^ when it was expanded in Taylor’s series in *k* and terms up to O (*k* ^4^) were retained. The derivation and the subsequent calculations were done employing the commercial package Mathematica™.

### Evaluation of Effective Slippage

The effective slippage $({b}_{eff})$ at the PS-PMMA interface was calculated employing the following procedure: (i) initially for a PMMA/PS/SiO_2_ configuration the dominant growth coefficient (${\omega }_{m}$) and wavelength (${\lambda }_{m}$) were evaluated from the GLSA employing the known physical properties and thicknesses; (ii) then, a small displacement, ${|{\tilde{w}}_{2}|}_{{h}_{20}}={\varepsilon }_{2}$, was assumed at the PMMA-air interface to convert the set of homogeneous boundary conditions to non-homogeneous set of algebraic equations; (iii) subsequently, the eight unknown constants, *B*_*ji*_ (*j* = 1 to 4 and *i* = 1, 2), were evaluated in terms of ${\varepsilon }_{2}$ employing any seven of the eight boundary conditions, which also helped in expressing the ${\tilde{u}}_{i}$, ${\tilde{w}}_{i}$ and ${\tilde{P}}_{i}$ in terms of ${\varepsilon }_{2}$; (iv) the tangential component of the hydrodynamic stress at the perturbed PS-PMMA interface, $({u}_{2z}+{w}_{2x})(1-{h}_{1x}^{2})+2{h}_{1x}({w}_{2z}-{u}_{2x})$ was linearized to, $d{\tilde{u}}_{2}={(-ik)}^{-1}{\tilde{w}}_{2zz}+ik{\tilde{w}}_{2}$; (v) finally, the effective slippage at the PS-PMMA interface was evaluated as, ${b}_{eff}={\tilde{u}}_{2}/d{\tilde{u}}_{2}$, replacing the values of ${\omega }_{m}$ and ${\lambda }_{m}$. The single layer linear stability analysis (SLSA) was performed employing the dispersion relation shown in the Eq. (8) of ref.^33^,

2 $$\rho {b}_{s}h{\omega }^{2}+\mu (4{b}_{s}{k}^{2}h+1)\omega +(h/3+{b}_{s})({\varphi }_{h}{k}^{2}{h}^{2}+\gamma {k}^{4}{h}^{2})=0$$

The SLSA plots were fitted against the GLSA after the following modifications: (i) we had to employ the interfacial tension of the PS-PMMA interface for the calculations, which indicated a dominant deformation of the interface than PMMA-air free-surface in the slip dominated instabilities of bilayers; (ii) the slip length had to be varied from non-slipping (10^−2^ μm) to strongly slipping (10^4^ μm) regime in order to match the predictions from the GLSA and experiments. However, this was evident only when the film thicknesses were kept constant and the viscosity of PS sublayer was varied.
